# Baby-Led Weaning vs. Traditional Complementary Feeding—Differences in Feeding Practices Among Polish Children Aged 6–36 Months—A Cross-Sectional Study

**DOI:** 10.3390/nu17050899

**Published:** 2025-03-04

**Authors:** Agnieszka Białek-Dratwa, Kinga Dawid, Wiktoria Staśkiewicz-Bartecka, Agata Kiciak, Oskar Kowalski

**Affiliations:** 1 Department of Human Nutrition, Department of Dietetics, School of Public Health in Bytom, Medical University of Silesia in Katowice, ul. Jordana 19, 41-808 Zabrze, Polandokowalski@sum.edu.pl (O.K.); 2 Department of Food Technology and Quality Assessment, School of Public Health in Bytom, Medical University of Silesia in Katowice, ul. Jordana 19, 41-808 Zabrze, Poland

**Keywords:** baby-led weaning, neophobia, complementary feeding, children’s diet

## Abstract

Complementary feeding involves introducing additional foods to a child’s diet, influenced by the child’s age, developmental stage, and skills, such as sucking, swallowing, and chewing. The WHO and ESPGHAN recommend exclusive breastfeeding for the first six months, with the gradual introduction of complementary foods thereafter. The baby-led weaning (BLW) method emphasises self-feeding and the early introduction of solid foods, fostering independence and development. This study aimed to evaluate the mode of complementary feeding in children aged 6–36 months, considering the BLW method. Material and Method: A cross-sectional survey was conducted among the parents of children aged 6–36 months using a Google Forms questionnaire distributed through social media. The study included 1027 participants, with 1017 mother–child pairs meeting the inclusion criteria. The questionnaire covered demographic data, feeding methods, and detailed questions on complementary feeding practices, including BLW. The study included children fed using the baby-led weaning method during supplementary feeding (BLW—baby-led weaning) and children not using the BLW method (NoBLW—no baby-led weaning). Results: This study found significant differences between the BLW and NoBLW groups in terms of feeding methods and outcomes. BLW children were more often exclusively breastfed (68.9% vs. 58.7%) and started complementary feeding later (79.9% between 6 and 7 months). Adverse events in the BLW group included gagging (64.8%), spitting out food (77.1%), and choking (12.3%), with rare cases requiring medical intervention (0.2%). Children using BLW showed greater autonomy in their eating decisions and had more frequent exposure to varied food textures. Conclusions: Children using the BLW method were more likely to be breastfed, had more contact with various textures, and were less likely to consume milk formula and pudding-type products. BLW, despite the risk of choking, is safe under supervision and supports motor development and healthy eating habits. The BLW method has the potential to support healthy eating habits and child independence, but its use requires parental education about safety and appropriate food choices. Further research should focus on the long-term effects of this method, especially in terms of its impact on children’s eating habits, motor development, and health at an older age.

## 1. Introduction

Complementary feeding (CF), as defined by the World Health Organization (WHO) in 2002, is “the process starting when breast milk alone is no longer sufficient to meet the nutritional requirements of infants” so that “other foods and liquids are needed, along with breast milk”. A child’s age, psychological and physical development, and skills significantly influence complementary feeding. The child’s skills, including sucking, swallowing, and grinding with the tongue or chewing, develop with age, allowing new food consistencies to be introduced. The WHO and the European Society for Paediatric Gastroenterology, Hepatology, and Nutrition (ESPGHAN) recommend exclusive breastfeeding for the first 6 months of life. The WHO supports the introduction of complementary foods from 6 months of age, while the ESPGHAN recommends their introduction no earlier than 4 months of age (week 17) and no later than 6 months of age (week 26). The WHO recommends breastfeeding up to two years and beyond [1,2,3,4].

CF is introduced to provide additional sources of energy, protein, iron, zinc, fat-soluble vitamins (A, D, E), and micronutrients and to prepare the baby for a more varied diet in the future. Due to their nutrient requirements, most infants need additional food beyond breast milk or milk mixtures at around 6 months of age. Most infants develop the ability to take solid foods between 17 and 26 weeks of age. During this time, babies acquire the ability to sit with support, control their head movements, and eat from a spoon. The reflex to push foreign bodies out of the mouth, which makes feeding with solid foods difficult, also disappears. The introduction of new foods does not mean that breastfeeding has to be stopped [1,4].

The introduction of complementary foods is necessary due to the baby’s growing energy and nutritional needs, which breast milk or milk formula alone cannot fulfil. This process should be gradual, starting with small amounts (three to four teaspoons) and monitoring the baby’s reactions. The type, consistency, and sequence of products introduced are the parents’ individual decisions. Complementary feeding also aims to prepare the child to eat whole foods in later years [5].

Most infants in Europe, including Poland, are not at risk of macronutrient deficiencies during dietary expansion. Data indicate that their energy, protein, sodium, and potassium intake are usually higher than recommended [4]. According to the ESPGHAN guidelines, parents choose the foods, and the child decides whether and how much to eat. The order in which foods are introduced is individual, and gradual feeding and repeated taste acceptance trials are key [4,6].

The WHO emphasises that infants in CF are at risk of malnutrition due to the inadequate nutritional value of foods, the inappropriate introduction of foods, and too early or infrequent breastfeeding [3]. To meet a child’s dietary needs, UNICEF recommends a varied diet, including breast milk, cereal products, vegetables, fruits, and animal and plant proteins. High-quality fats, especially omega-3 fatty acids, to support cognitive and physical development are also key. Low-nutrition foods such as sweets and sweetened drinks, which can promote obesity, should be avoided [7].

The baby-led weaning (BLW) method is based on the baby’s self-management of the feeding process, promoting the early introduction of solid foods and breastfeeding. The key to this method is to consider the child’s appetite and create the right atmosphere at mealtimes, without forcing them to eat. BLW is intended as an adjunct to breastfeeding or formula milk and not a replacement. The recommendations of the Polish Society for Paediatric Gastroenterology, Hepatology, and Nutrition (Polskie Towarzystwo Gastroenetrologii, Hepatologii i Żywienia Dzieci—PTGHiZD) indicate that BLW should be started between the sixth and seventh months of a child’s life, omitting the use of spoons and purees in favour of products that the child can quickly grasp [1,8].

The complementary feeding method, BLW, was adapted to create the innovative baby-led introduction to solids method (BLISS). BLISS is designed to potentially enhance the baby’s development compared to the BLW method. Parents using BLISS introduce energy- and iron-rich foods, such as meat, fish, and iron-fortified foods, which could offer significant nutritional benefits. However, it is important to note that there is currently a lack of information about whether the BLISS method has beneficial or harmful effects on the child’s health [1,5,6,9,10,11].

One risk of using the BLW method is the possibility of the baby gagging on solid foods. These include foods that can pose obstacles in the respiratory tract, such as grapes, peas, sliced sausages, roasted corn, chewing gums, nuts, raw carrots, and apples. Parents should also refrain from using small, hard foods with smooth surfaces to minimise the risk of gagging. It is important for parents to be aware of the potential risks of the BLW method. In the case of children, food is often spat out just before gagging occurs. Although there have been isolated cases of gagging in the literature reviewed, there are still concerns among parents about using the BLW method [5,12,13].

Food neophobia is a reluctance to eat and try new foods, which can lead to a monotonous diet and nutrient deficiencies. This can negatively affect a child’s health, growth, and quality of life. Infants accustomed to the taste of milk may be reluctant to accept new foods, so it is important to gradually introduce different tastes in a complementary diet [1,14,15,16]. The causes of neophobia are diverse—cultural, environmental, and evolutionary. Treatment should be team-based and involve a paediatrician, nutritionist, psychologist, neurologist, and therapist. The complete BLW method may reduce the risk of this problem [17,18,19,20].

This study aimed to evaluate the mode of complementary feeding in children aged 6–36 months, considering the BLW method. It also included an assessment of how children were fed before starting complementary feeding, how new foods were introduced into the child’s diet, and a comparison of the traditional complementary feeding method with the BLW method.

## 2. Materials and Methods

### 2.1. Research Tool

The questionnaire was created using the Google Forms application. This study was conducted by the questionnaire method, using an indirect survey technique through computer-assisted web interviewing (CAWI). The survey was conducted using the CAWI method. Respondents completed the survey electronically through community groups for the parents of babies and young children. According to Polish law, the mother, after giving birth, is entitled to 20 weeks of maternity leave if she gives birth to one child, 31 weeks for twins, 33 weeks for triplets, 35 weeks with four children born at the same time, and 37 weeks if five or more children are born during one birth [21]. After maternity leave, both parents can take parental leave, which amounts to 32 weeks in the case of one child, giving almost a year of being at home with the child, without having to return to work. Data from the Social Insurance Institution show that, from January to May 2021, more than 246,000 parents received maternity benefits for the period of parental leave, of which only 1900 were men, the remainder of more than 244,000 were mothers [22]. At the same time, there are thousands of groups on social media that bring together Polish mothers from different cities, towns, and villages in Poland, with children who were born in a given month and in a given year. In the present study, groups of mothers that referred to feeding were not considered, so that the results of the study would not be biased by mothers who were more interested in the complementary feeding of their children.

This study used a recruitment method based on the snowball effect, whereby each participant was asked to pass the questionnaire on to other potential respondents. All study participants were informed about the purpose of the study, the voluntary nature of their participation, and the anonymity of the presentation of the study results. At the beginning of the CAVI questionnaire, they were asked to accept the data sharing rules and gave informed consent to participate in the study. The survey was conducted between November 2023 and March 2024. The study was conducted in accordance with the Declaration of Helsinki and the Act on the Profession of Physicians and Dentists. A positive opinion was obtained from the Bioethics Committee operating at the Silesian Medical University in Katowice to conduct a study on parents’ knowledge of young children’s nutrition (2022) (PCN/CBN/0052/KB/101/22).

### 2.2. Study Group

**Figure 1:**
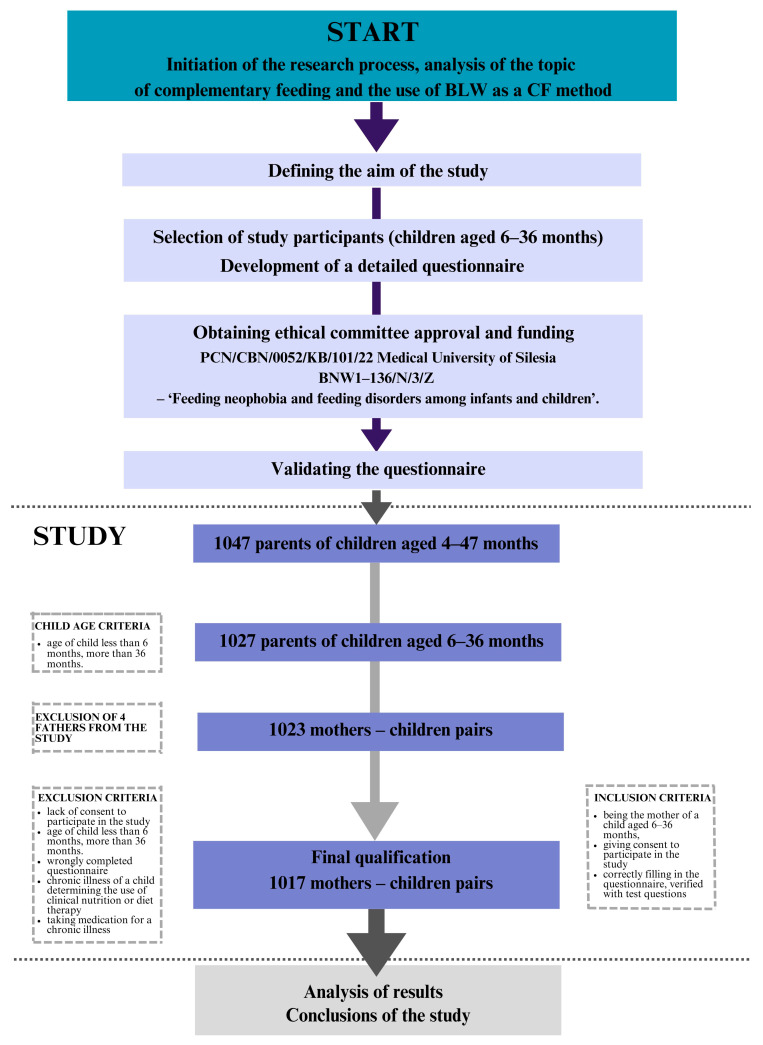
Study design.

A survey was conducted on how infants and young children are fed. The survey was aimed at the parents of children aged 6–36 months. The study group consisted of 1027 parents aged 19–51 years. Inclusion and exclusion criteria were included in the study. The inclusion criteria were being a parent of a child aged 6–36 months, consenting to participate in the study, providing informed consent, and correctly completing the questionnaire, as verified by test questions. The criteria for exclusion from the study were being a parent of a child younger than six months and older than 36 months and an incorrectly completed questionnaire. Men were excluded from the compiled results due to the low turnout of the survey group, with N = 4 respondents (0.4%). Conditions affecting feeding, such as food allergies or intolerances or autism spectrum disorder, as well as nutritional treatment and ongoing medication, were included in the exclusion criteria. After considering the inclusion and exclusion criteria, 1017 mother–child pairs were included in the final analysis. The number of women of childbearing age in Poland in 2023 was 8.7 million. We therefore used a representative sample size calculator, with an estimated error of 5%. Our sample population size was required to be a minimum of 385 women. In our study, we increased the sample size by almost three times to minimise research errors (Figure 1).

This study identified two sub-groups of children: children fed by the BLW method (BLW) and those not fed by the BLW method (NoBLW). Assignment to the respective group was based on multiple verifications. The basis for qualification was the mother’s assessment of whether she used the BLW method during CF. Additionally, it was assessed whether the child had the possibility to feed themselves during CF by eating with their hands or by eating with cutlery such as a spoon/fork. Based on the analysis of the above questions, 586 children (57.6%) were selected in whom the BLW method was used and 431 (42.4%) in whom only the adult method of feeding the child with a spoon/fork (NoBLW) was used.

### 2.3. Survey Questionnaire

For the purposes of this study, a questionnaire was developed, consisting of 22 questions, addressing different aspects of infant feeding, including demographic information, feeding practices during the first six months of life, and complementary feeding.

The first section of the questionnaire focused on demographic data, such as the parents’ age, education, and place of residence, as well as basic information about the child, including their age, weight, and height. Using these data, along with percentile charts and BMI-for-age standards for children aged 0–3 years, the child’s weight status was assessed as underweight, normal weight, overweight, or obese, according to the WHO standards [23,24]. In this study, the World Health Organization (WHO) reference centile charts were used to assess the body mass index (BMI) of children aged 0 to 36 months. BMI data and body mass classifications were assigned based on the child’s age and sex, taking into account the reference values for each age group. The calculated values were compared with the WHO centile ranges. Based on these values, the following classification was adopted: BMI below the 3rd centile—underweight, BMI from the 3rd to the 85th centile—normal weight, BMI from the 85th to the 97th centile—overweight, BMI above the 97th centile—obesity. Information on the child’s current weight and height was obtained from the “Child Health Booklet”, which serves as an official medical record in Poland [25]. This part of the questionnaire also considered the child’s chronic illnesses and any medication that the child took regularly.

The second part of the questionnaire concerned feeding practices during the first six months of the child’s life, including questions about exclusive breastfeeding, its duration, and the use of formula milk. Respondents were asked to indicate for how long their child was breastfed and when they introduced the formula, which allowed for a detailed analysis of infant feeding patterns. The rest of the questionnaire included questions about the timing of the introduction of complementary foods, the consistency of meals (purees, finger foods), and the types of products introduced, such as vegetables (carrots, potatoes, pumpkins), fruit (apples, pears), meat (poultry, beef, lamb), fats (rapeseed oil, olive oil), and cereals (wheat, rice, oats). We considered the timing of introducing the above-mentioned foods into the child’s diet, categorised as follows: before 4 months of age, between 4 and 6 months, between 6 and 12 months, after 12 months, not introduced, or not remembered. Detailed questions in the form of tables made it possible to determine when the individual food groups were introduced, which allowed for an analysis of the compliance of the feeding practices with the recommendations.

The questionnaire also included questions verifying the method of complementary feeding, which made it possible to distinguish between children fed with a spoon and those fed using the baby-led weaning (BLW) method. For parents using BLW, questions were included about the time of initiation, the types of products fed, feeding experiences (e.g., gag reflex, choking risk), and the sources of knowledge about BLW, such as consultations with a dietitian or paediatrician or information obtained from social media. In addition, the survey included questions about the advantages and disadvantages of the BLW method, which allowed us to learn about parents’ opinions of this method. Precise definitions of the terms used, such as “exclusive breastfeeding” or “BLW”, and different types of questions (single- and multiple-choice and tabular questions) provided accurate data for the analysis of the children’s eating habits, taking into account the time aspect, qualitative aspects, and the subjective assessments of the parents.

The questionnaire was developed based on the current nutritional recommendations for infants and young children, as outlined by the PTGHiŻD [1], based on the ESPGHAN guidelines [4], and on information about the BLW method [26,27,28,29,30,31,32,33,34,35]. The survey also included more detailed questions, such as whether the child experienced choking or gagging incidents that required medical intervention.

A pilot study was conducted on a group of 30 mothers to validate the questionnaire and assess the relevance of the questions. The reproducibility of the responses was checked after one month by calculating the Kappa coefficient (ϰ). Very good agreement (ϰ ≥ 0.80) was obtained for 71.2% of the questions, good agreement (0.79 ≥ ϰ ≥ 0.60) for 22.5%, and moderate agreement (ϰ < 0.59) for 6.3%. The Cronbach’s alpha coefficient was 0.86, confirming the high reliability of the questionnaire.

### 2.4. Statistical Analysis

Statistical tests were used for the statistical analysis, which was performed using Statistica v. 13.3 (StatSoft Inc., Tulsa, OK, USA). The descriptive statistics in the data evaluation included numbers. The Kolmogorov–Smirnov test checked the alignment of the variables with a normal distribution. The data did not have a normal distribution, so non-parametric tests were subsequently used. The Mann–Whitney U test was used to compare mean values. Median values were assessed for the comparison of the weight, age, and height in the BLW/NoBLW groups of children studied. The chi-squared was used for statistical calculations to compare the differences between the BLW-using and NoBLW-using groups. Cramér’s V and Phi coefficients were used to analyse the strength of the relationships among the qualitative variables. For continuous variables, the strength of the effect was determined by the coefficient r. The interpretation of the strength of effect for Cramér’s V (Cr.V), Phi, and r was based on standard thresholds: 0.1—weak dependence, 0.3—moderate dependence, 0.5—strong dependence. Results were assessed with a 95% confidence interval, and the level of statistical significance was set at *p* <.05.

Two regression models were used to assess the impact of baby-led weaning (BLW) on children’s weight while controlling for age differences between the groups: linear and logistic regression. The linear regression model was used to examine the relationship between weight and BLW while considering the child’s age as a covariate. A logistic regression model assessed the risk of being outside the normal body weight range (underweight or overweight).

## 3. Results

### 3.1. Characteristics of the Study Group

There were no significant differences in the use of the BLW method according to the mothers’ educational levels (*p* = 0.509). BLW was used by 69.3% of mothers with a tertiary education, 27.3% with a secondary education, 2.6% with a vocational education, and 0.9% with a primary education. Similarly, there were no significant differences in BLW use according to the child’s gender (*p* = 0.0702): boys accounted for 49.7% of BLW users and girls for 50.3%. The median age of children using BLW was 16 months, significantly higher than in the group of children not using BLW (13 months, *p* < 0.0001). The body weight of children using BLW was significantly higher (median 10.5 kg) than that of children with NoBLW (median 10.0 kg, *p* = 0.0022). The height of children using BLW was also greater (median 82 cm) compared to children with NoBLW (median 80 cm, *p* < 0.0001) (Table 1).

### 3.2. Infant Nutrition During the First Six Months of Life and During Complementary Feeding

The statistical analysis showed significant differences between children using the BLW method and children with NoBLW in terms of the feeding method in the first months of life and the length of breastfeeding. Children using BLW were more often exclusively breastfed (68.9%) than children not using BLW (58.7%), which was statistically significant (*p* = 0.0007). In contrast, feeding with milk formula was less frequent in the BLW group (59.7%) than in the NoBLW group (71.7%), which was also statistically significant (*p* = 0.00008). Regarding the length of breastfeeding, children using BLW were more likely to be breastfed for longer, with 35.7% continuing to be breastfed, compared to 29.9% of children with NoBLW (*p* = 0.000). There was no significant difference between the groups in the timing of milk formula introduction, referring to the age at which it was first introduced (*p* = 0.07). In contrast, the initiation of complementary feeding differed statistically (*p* = 0.006), with most children using BLW starting complementary feeding between 6 and 7 months of age (79.9%, compared to 74.5% in the NoBLW group) (Table 2).

The introduction of the first foods during the start of complementary feeding, such as green vegetables, pumpkin, carrots, potatoes, porridge, apples, and pears, showed no significant differences between the groups. Carrot was the most frequently introduced product in both groups: 41.3% of children using BLW and 45.0% of children NoBLW, for a total of 42.9% (Table 3).

In the group of children with BLW, 89.8% were introduced to foods with lumps, such as fruit purees with small pieces or yogurts with added ingredients, compared to 95.34% in the NoBLW group (*p* = 0.001). This statistically significant difference suggests that, while both groups had exposure to more complex food textures, children in the NoBLW group were more frequently introduced to such foods. The introduction of lumpy foods is crucial as it aids in the development of oral motor skills and teaches children to handle a variety of food textures. Additionally, 98.8% of children in the BLW group received foods that they could grasp and feed themselves, compared to 84.2% in the NoBLW group (*p* = 0.000). This statistically significant difference highlights that the BLW method places a stronger emphasis on encouraging children to self-feed from the beginning. BLW children are more inclined to engage with foods that they can physically handle, which supports their independence and the development of fine motor skills, such as precise grasping and controlling portion sizes (Table 3).

In the analysis of the introduction of complementary foods, significant differences were found between children fed using the BLW method and those fed traditionally. Vegetables were introduced most frequently between 6 and 12 months of age in 65.9% of BLW children and 55.2% of NoBLW children (*p* = 0.006). Similarly, fruit was introduced during the same period in 69.1% of BLW children and 58.5% of NoBLW children (*p* = 0.01). Eggs were introduced between 6 and 12 months of age in 80.4% of BLW and 75.6% of NoBLW children (*p* = 0.003). Meat and fish were introduced similarly in both groups, in 78.8% of BLW and 75.6% of NoBLW children, respectively (*p* = 0.73). Gluten was introduced more frequently in the BLW group (79.9%) than in the NoBLW group (72.2%, *p* = 0.0005), as were cereal products (77.6% vs. 68.4%, *p* = 0.00003). Peanuts were introduced more frequently in the BLW group (44.2%) than in the NoBLW group (32.7%, *p* = 0.0004). As for water, BLW children were more likely to have it introduced between 6 and 12 months of age (59.9% vs. 49.0%, *p* = 0.006) (Table 4).

### 3.3. Complementary Feeding Among Children Using the BLW Method

The use of the BLW method was associated with adverse events such as the vomiting reflex (47.3%), spitting out food (77.1%), gagging (64.8%), and choking (12.3%). Only 0.2% of children required medical intervention due to choking. The BLW method was most commonly introduced between 6 and 7 months of age (60.6%) and, to a lesser extent, between 4 and 5 months (2.7%), 8 and 12 months (35.0%), and after 12 months of age (1.7%). The BLW method promotes a child’s autonomy in their feeding habits. When using this method, 92.7% of children could decide what they would eat and 99.8% how much they would eat. This empowerment is a significant benefit of the BLW method. In addition, 81.6% of children were spoon-fed, 70.7% ate independently with a spoon, and 98.1% ate independently with their hands (Table 5).

Table 6 presents the results of two regression models evaluating the association between baby-led weaning (BLW), the child’s age (in months), and weight-related outcomes. The linear regression model predicts body weight (kg) as a function of BLW and the child’s age, while the logistic regression model predicts the likelihood of having a healthy weight status (normal weight vs. underweight/overweight/obesity). BLW is treated as a binary variable (1 = BLW, 0 = non-BLW). The beta coefficients (β) represent the effect size of each predictor, with *p*-values indicating statistical significance. Confidence intervals (95% CI) provide a range of plausible values for each estimate. The results indicate that the child’s age was a significant predictor of body weight (β = 0.227, *p* < 0.001), with an average weight increase of 0.227 kg per month. However, BLW had no significant effect on body weight (β = −0.026, *p* = 0.758). The linear regression model explained 50.2% of the variance in body weight (R^2^ = 0.502). In the logistic regression model, neither BLW (β = 0.071, *p* = 0.595) nor the child’s age (β = 0.019, *p* = 0.097) were significant predictors of a healthy weight status. The model had low explanatory power (Pseudo R^2^ = 0.0026), suggesting that the included variables did not adequately predict whether a child fell within the healthy weight range. These findings indicate that, while age is a strong predictor of body weight, BLW does not significantly influence body weight or the likelihood of being within a healthy weight range.

## 4. Discussion

### 4.1. Feeding in the First Months of a Child’s Life

The data analysis showed that 657 (68.9%) women exclusively breastfed their children. In a study by Bialek-Dratwa et al. [13], 306 (47.7%) women confirmed their use of exclusive breastfeeding until their child was six months old, and 109 mothers (16.87%) continued breastfeeding until their child was one year old. In addition, more than 30% of mothers continued to use natural feeding. In our study, similar results were obtained: 33.2% of mothers continued to breastfeed, while only 10% stated that they breastfed their child until the child was 12 months old. The total result of almost 70% confirms that more women are opting for the currently promoted breastfeeding. Królik-Olejnik et al. [36] included 43.5% of children exclusively breastfed up to 2 months of age; in their study, a result of 8% was reported. They also considered the report entitled, “Evaluation of the implementation of lactation practices within the current standard of perinatal care and feeding children from birth to 12”. The following results concerning breastfeeding were noted: on the day of discharge from the hospital, 75% of respondents were breastfeeding; by 2 months of age, 72.48% were breastfeeding; exclusive breastfeeding was noted in 43.5%; in the next stage of the study, at up to 4 months of age, 41% of respondents confirmed the use of breastfeeding regarding the baseline group; in the next stage, at up to 6 months of age, only approximately 39% were breastfeeding mothers; meanwhile, in the last stage of the study—up to the 12th month of life—only 17% confirmed their use of breastfeeding [36,37].

### 4.2. Complementary Feeding

Our study showed that most mothers chose to start complementary feeding with their baby at between 6 and 7 months of age, in line with the WHO recommendations. The Polish and European recommendations suggest introducing complementary products slightly earlier, at week 17, i.e., at the beginning of the child’s fifth month of life. In the study by Białek-Dratwa et al. [13], almost 50% of the respondents who did not use the BLW method declared that they started complementary feeding at between 4 and 6 months of the child’s life. However, in the BLW group, 63.68% of the respondents introduced solid foods after 6 months. In the Kozioł-Kozakowska et al. [38] study, nearly 50% of the parents surveyed started complementary feeding between 17 and 26 weeks. The study by Zielińska et al. [39] reported a higher percentage who introduced complementary foods between 4 and 6 months of age, at 65%; after 6 months of age, it was 32.1%; and before 4 months of age, this was only 3% of the respondents.

Research indicates that the early experience of consuming different types of food can significantly influence the development of a child’s food preferences in later years. In particular, the development of taste acceptance of vegetables is more challenging than that for fruit, suggesting the need for the early introduction of vegetables into an infant’s diet. Vegetables, especially greens, are more difficult to accept due to their specific taste, so it is recommended to introduce them before fruit. Naturally sweeter and more accessible to accept, fruits should be added to the diet about two weeks after the introduction of vegetables, while continuing to give vegetables. Such a regimen can foster the development of long-term vegetable acceptance in the child and support balanced eating habits [4].

Regarding the first product introduced into the child’s diet, our study found a result of more than 40% for carrots. A study by Malczyk et al. [40] reported that 95.1% of the respondents introduced carrots, potatoes, and pumpkins to their child’s diet. In contrast, in our study, more respondents chose green vegetables over potatoes, which may indicate the higher nutritional awareness of mothers when expanding their children’s diets. In addition, a larger proportion of mothers using the BLW method introduced green vegetables first. In a study by Koziol-Kozakowska [38], the largest percentage of parents (60.6%) introduced carrots as the first vegetable. Moreover, in Kostecka’s study, pureed carrots appeared to be the first product (83%) [41].

Analysing the subsequent foods in terms of the timing of the introduction of each product in the study by Malczyk et al. [40], 41% of parents introduced vegetables between 5 and 6 months of age, and 45.9% of respondents introduced fruit between 5 and 7 months. In our study, the results were slightly lower, with 36.8% for vegetables and 33% for fruit introduced between 4 and 6 months of age. Malczyk et al. [40] reported that 40.6% of parents introduced eggs between 7 and 9 months of age; in our study, the result was much higher, with 78.4% of parents declaring the introduction of eggs between 6 and 12 months of age. The PTGHIŻD recommendations suggest introducing eggs at least two times a week during CF, boiled or not fully cooked, in the form of pancakes or scrambled eggs [1]. The data analysis by Caffarelli et al. [42] showed that delaying the introduction of allergenic products, i.e., eggs and peanuts, does not reduce the child’s risk of allergy. In high-risk children, the introduction of allergenic products before 4 months of age has also not been shown to reduce the risk of allergy [43,44].

Our study found that processed meats, which should not be introduced into children’s diets before the age of 3 years, were introduced by nearly 40% of mothers between 6 and 12 months of age. However, it is encouraging to note that approximately 20% of respondents introduced them after 12 months of age, while more than 30% of mothers did not introduce them at all. This high percentage of mothers who did not introduce processed meats is a positive indication of healthy feeding practices. In a study by Malczyk et al., nearly 27% of respondents introduced meat products at between 7 and 9 months of the child’s life [40]. Young children are not recommended to consume processed meat due to its high salt content, additives such as nitrates and nitrites, and low nutritional value. Studies indicate that excessive salt intake can strain developing kidneys, and compounds formed from preservatives can be potentially carcinogenic [1,2,3,4].

### 4.3. Complementary Feeding and the Introduction of the BLW Method

When comparing the use of the traditional and BLW feeding methods, Zielinska et al.’s study reported a slightly higher percentage of mothers using BLW than in their own study (69.3%; 57.6%) [39]. Regarding the use of the BLW method in their own study, 87.2% of the mothers declared the introduction of pudding, similar to the 2022 study (Białek-Dratwa et al.), where 76.76% of the respondents declared similar practices. In the case of lumpy pudding, the result obtained in the self-study was close to 322 90%, while, in the 2022 self-study, it was around 75%. Although the majority of mothers used the BLW method, the large percentage of those who used pudding with lumps suggests incomplete BLW [13].

When analysing the risk of choking, our study reported 277 cases (47.3%) of the vomiting reflex, which was higher than in the study by Białek-Dratwa et al., where 34.9% of cases were reported. It is important to note that parents using the BLW method are often concerned about their children choking on food [17]. In our study, choking was reported in 72 cases (12.3%), while the 2022 study reported 7% of cases. In both our study and the 2022 study, a small proportion of cases (0.2% and 0.45%, respectively) required medical assistance [13]. A meta-analysis by D’Aurii [45] analysed the risk of choking during the baby-led weaning (BLW) method in infants learning to eat independently. At the onset of complementary feeding, around 6 months of age, the child may not yet have the developed oral motor skills, such as chewing and swallowing, necessary to consume whole foods safely. Not all children are ready to be introduced to solid foods at 6 months of age or earlier [43,46,47]. A study by Townsend E. [44] found no difference in choking rates between the BLW method group and children who were traditionally spoon-fed. A study by Cameron SL [48] found that 30% of children fed using the BLW method had at least one choking episode after eating solid food. Similar results were obtained in a study by Brown [30], which included 1151 infants and looked at the risk of choking and whooping. The results indicated that at least one choking episode occurred in 11.9 per cent of children in the BLW-fed group, in 15.5 per cent of children in the “loose BLW” group, and in 11.6 per cent of children who were traditionally spoon-fed, with no significant differences between the groups. In a study by Quintiliano-Scarpelli D. and colleagues, the incidence of the vomiting reflex, choking, and suffocation was 78.2%, 28.4%, and 3.1%, respectively [49].

Vomiting, especially in the context of the BLW method, may be the result of the rapid introduction of inappropriate food textures or insufficient supervision. Although only 0.2 per cent of cases in this study required medical intervention, the frequency of vomiting and choking reflexes, which occurred in 47.3 per cent of children using the BLW method, may also be an important risk indicator. Food choices should be matched to the child’s ability, and parents need to introduce foods gradually, monitoring the reactions to individual foods. Thus, the frequency of vomiting may also be an indicator that certain foods or methods of serving them are not yet suitable for the child.

Significant differences were also observed in the aspect of spoon feeding. In our study, 70.7% of the respondents stated that the child ate independently with a spoon; in the 2022 study, this percentage was 29.06%. On the other hand, the spoon feeding of the child by the parent was reported in more than 80% of cases in our study, while, in the study by Białek-Dratwa et al., this was 6.3% [13].

Practising BLW or spoon feeding does not have to occur exclusively as a choice between one or the other. Research shows that many families use a mixed approach, combining independent feeding (BLW) with spoon feeding. In the study group, 81.6 per cent of children fed using the BLW method were spoon-fed, suggesting that these methods can be used in parallel. Parents often start with spoon feeding and switch to BLW or use the two methods, depending on the situation and the child’s readiness. Adapting the method to the child’s individual needs and development and parental preferences is important.

In the standards of the Polish Society of Gastroenterology, Hepatology, and Child Nutrition, one recommendation for CF reads as follows: “The parent/guardian decides what the child will eat and when and how the food will be served. The child decides whether to eat the meal and how much he/she will eat” [1]. In the BLW method, one of the assumptions is that the child can decide for themselves how much they can eat and what they can eat from the products served. This aspect of the BLW method, which promotes the child’s autonomy in their food choices and intake, was evident in our study. Almost 100% of the children could decide how much they wanted to eat and around 90% chose what they could eat. In another self-reported study in 2022, the results were reported to be similar (93.22%) regarding how much the child wished to eat, and the result was slightly lower regarding what the child wished to eat (65.62%) [13].

Eating independently using the BLW method promotes the development of motor skills and independence and contributes to building a better awareness of food and satiety. However, to promote healthy growth and weight gain, adequate variety in the diet and the appropriateness of the foods introduced are equally important. It is emphasised that children should be provided with foods rich in energy, protein, fat, and micronutrients such as iron, zinc, and vitamins A and D to ensure a complete diet. Dietary compliance with these guidelines is crucial for proper physical development. BLW allows children to explore different textures and flavours, which can support greater dietary diversity. However, this study found that the variety of foods introduced depends on the parents’ practices and that care should be taken to ensure that the diet is well balanced.

The analysis of the study results allows us to identify three critical aspects regarding complementary feeding: the method of food introduction (BLW vs. traditional feeding), the age at the initiation of complementary feeding, and dietary diversity practices. As shown in this study (Table 3), regardless of the method used, most children started complementary feeding between 6 and 7 months of age, aligning with the WHO recommendations. However, children using the BLW method were likelier to have independent food choices. They were introduced to more varied food textures, which supported their independence and the development of motor skills such as precise grasping and food manipulation.

### 4.4. Introduction of Food Groups and WHO and ESPGHAN Recommendations

BLW children were more exposed to various foods, such as vegetables, fruit, gluten, and cereal products, than traditionally fed children. These results suggest that BLW may promote the early acceptance of various tastes and textures, essential for the development of healthy eating habits later in life. The introduction of vegetables, especially greens, and fruit, between 6 and 12 months of age aligns with the current WHO and ESPGHAN recommendations, emphasising the importance of early dietary diversity for healthy child development.

A critical aspect of the proposals is the difference in introducing gluten- and peanut-containing products. The early introduction of gluten in the diets of BLW children (79.9 per cent between 6 and 12 months of age) may align with research findings that suggest that the moderate introduction of this ingredient at an appropriate time reduces the risk of gluten intolerance. Similarly, peanuts were introduced more frequently in the BLW group, which may indicate greater parental awareness of the benefits of the early introduction of potential allergens. The literature highlights that avoiding the delayed introduction of allergens may reduce the risk of food allergies.

The results regarding introducing water as the primary liquid after 6 months of age (59.9% in the BLW group) emphasise that this method can promote healthy habits, in line with the recommendations of the PTGHiŻD and WHO. Unlike sweetened drinks, water promotes a healthy fluid balance and reduces the risk of excessive sugar intake in the first years of a child’s life. In contrast, the introduction of honey in some children before 12 months (9.0% in the BLW group, 10.2% in the NoBLW group) indicates a lack of complete adherence to the recommendations, and this may increase the risk of infant botulism. This result indicates the need to further educate parents about the risks of introducing certain products.

The introduction of salt in the diets of BLW children (19.8 per cent between 6 and 12 months of age) and sweetened beverages (9.2 per cent during the same period) remains a challenge despite the current guidelines recommending their avoidance in the first two years of life. High salt content can negatively affect the development of a child’s taste preferences, increasing the risk of hypertension in adulthood. Excessive sugar consumption, on the other hand, is linked to the risk of overweight and obesity, as well as tooth decay.

The BLW method, by promoting the child’s independence in food choice, can positively impact the development of motor skills, the awareness of one’s own nutritional needs, and one’s long-term eating habits. However, the success of this method largely depends on educating parents on the appropriate choice of foods, monitoring their child’s reactions, and avoiding products with potential risks, such as honey or processed meat. It is also worth noting that BLW promotes greater dietary diversity, which can counteract monothematic diets and nutrient deficiencies.

In conclusion, the BLW method shows significant advantages in terms of promoting healthy eating habits and dietary diversity. However, it is essential to support parents through nutritional education to reduce the risks associated with the introduction of inappropriate products and ensure a wholesome diet for the child.

The discussion of these results indicates that the BLW method promotes the child’s independence and sensory–motor development by allowing the child to actively participate in the eating process. In contrast, the traditional method (spoon feeding) provides a more controlled means of introducing new foods, which may be beneficial for children who need more support in developing their eating skills. The results suggest that both methods can be used in parallel, offering a flexible approach that empowers parents and healthcare professionals to tailor feeding to the individual child’s needs.

In the context of the recommended dietary diversity, it is crucial that, whatever method is chosen, richness of flavours and textures is promoted, which not only supports physical development but also contributes to the acceptance of different foods at a later age. This study highlights the importance of parents’ role in monitoring the child’s response to the foods introduced and adjusting the rate and type of complementary feeding according to the child’s developmental needs, making them feel responsible and attentive.

## 5. Limitations and Strengths of the Study

This study has strengths and limitations, which should be considered when interpreting the results. One of the main strengths is the large sample size (1027 parents), which enhances the representativeness and provides a broad overview of CF practices and the use of BLW in real-world settings. The anonymous and voluntary nature of the study likely increased the likelihood of honest responses, thereby improving the data quality. The questionnaire included questions on demographics, complementary feeding, the BLW method, and detailed inquiries regarding adverse events such as choking and gagging, allowing for comprehensive data collection.

The study’s reliance on parental self-reports enabled us to capture feeding practices in natural home environments, which is particularly important since most feeding practices, such as breastfeeding and formula feeding, occur at home, rather than in controlled clinical settings. The questionnaire was designed according to the latest guidelines from the PTGHiŻD and ESPGHAN, ensuring alignment with international standards. Moreover, the study covered various aspects of feeding, including breastfeeding, formula feeding, the introduction of complementary foods, and the occurrence of adverse events, which allowed for a comprehensive assessment of feeding practices.

However, significant limitations to the study must also be considered. First, as a cross-sectional study based on a single survey, it did not allow for the tracking of long-term outcomes related to different feeding methods, such as BLW or traditional feeding. Longitudinal studies or randomised controlled trials could provide more reliable and generalisable conclusions. Another area for improvement is the snowball sampling method, which may lead to a non-representative sample, since respondents might recommend the survey to individuals with similar demographic characteristics or views. While the computer-assisted web interviewing (CAWI) method has several advantages, it limits the sample to individuals with internet access and technological familiarity, potentially introducing bias.

Additionally, the study relied on parental declarations, which carry the risk of subjectivity and reporting bias, particularly regarding adverse events such as choking and gagging, as well as the assessment of food diversity and texture. The need for the more objective verification of these data is a significant methodological limitation. Furthermore, this study did not account for confounding variables such as educational levels, access to lactation support, or socioeconomic conditions, which may influence parental feeding practices. The absence of the direct observation of feeding practices also limits the accuracy of the results, although clinical observation is not feasible for this type of study.

In conclusion, this study provides valuable insights into complementary feeding practices and the use of BLW in home environments. However, methodological limitations indicate the need for more rigorous research in the future. Despite its limitations, the research tool developed for this study offers a better understanding of feeding practices among the parents of young children. This study represents an essential step towards a deeper understanding of modern feeding practices and may contribute to future clinical recommendations supporting healthy child development.

## 6. Conclusions

Children using the BLW method were more often exclusively breastfed for longer than children not using this method. Feeding with milk formula was less frequent in the BLW group than in the NoBLW group. The timing of the introduction of milk formula showed no significant differences between the groups. At the same time, the initiation of complementary feeding was statistically different, as a larger proportion of children using BLW started complementary feeding between 6 and 7 months of age. The use of pudding and pudding with lumps was less common in the BLW group than in the NoBLW group.

The BLW method was often introduced between 6 and 7 months of age, suggesting that this is the optimal time to start. Children using BLW often had the opportunity to decide on the type and amount of food that they ate, which can support the development of independence and motor skills.

Before the introduction of BLW, the majority of children were spoon-fed, and, after its introduction, the children ate independently with a spoon; at the same time, children using BLW have more frequent contact with a variety of textures and forms of food, which can support their motor development and independence in eating. The BLW method allows children to be more independent in their choices and consumption of food, while requiring adequate supervision due to the risk of choking.

The BLW method carries some risk regarding the vomiting reflex, spitting out food, gagging (64.8%), and choking (12.3%), but cases requiring medical intervention are rare (0.2%). Although there is a risk of gagging, in general, the BLW method is safe with appropriate adult supervision.

The BLW method had no significant effects on children’s body weight or the likelihood of having a healthy weight. The child’s age had a significant effect on their body weight but did not significantly affect the likelihood of being within a healthy weight range.

Introducing a variety of products in line with the current dietary recommendations is crucial for the healthy development of children.

## 7. Practical Dietary Implications

Based on the present study, it is possible to formulate practical dietary implications concerning the introduction of complementary foods using the baby-led weaning (BLW) method.

1. The BLW method, a beneficial approach, is best introduced between 6 and 7 months of a child’s life. At this age, children usually have developed motor and sensory skills that enable them to eat independently, fostering a sense of confidence and self-reliance.
2. Complementary foods should be introduced gradually, starting with small portions and monitoring the baby’s reactions. It is advisable to introduce vegetables (especially green vegetables—they accustom children to a bitter taste) before fruit to support the development of an acceptance of more challenging tastes.
3. The BLW method allows the child to choose and control the amount of food that he or she eats independently, which promotes the development of independence, motor skills, and an awareness of the body’s needs.
4. Introducing a variety of foods, such as vegetables, fruit, meat, eggs, fish, and cereal products, is key to ensuring an adequate supply of energy, proteins, fats, and micronutrients. BLW encourages a greater variety of textures and tastes.
5. Special care should be taken when using BLW to minimise the risk of choking. It is recommended to avoid hard, small, and slippery foods (e.g., grapes, nuts) and to ensure adult supervision during meals.
6. The consistency and form of foods should be adapted to the child’s age and ability. Introducing lumpy foods at the right time supports the development of chewing and swallowing skills.
7. Combining BLW with traditional spoon feeding can benefit families who wish to adapt the feeding method to their child’s needs and their family situation. This combination can provide a balanced approach, allowing the child to explore and develop their independence with BLW, while also ensuring that they receive adequate nutrition through spoon feeding.
8. Parents play a crucial role in this process. They should carefully observe their child’s reactions to the products introduced and respond to any signs of intolerance, allergy, or eating difficulties, ensuring their child’s safety and well-being.
9. Parents should be educated about the principles of the BLW method, its risks, and it benefits and empowered to follow the current dietary recommendations for children, which can improve the quality of the introduction of complementary foods and their child’s overall health.

## Figures and Tables

**Table 1:** Characteristics of the study group of mothers and their children.

	BLW	NoBLW	Total	*p*-Value
Place of residence				
City	390 (66.6%)	260 (60.3%)	650 (63.9%)	*p* = 0.0409 * Cr.V = 0.064
Village	196 (33.4%)	171 (39.7%)	367 (36.1%)	
Mother’s education				
Basic	5 (0.9%)	2 (0.5%)	7 (0.7%)	*p* = 0.509 *
Professional	15 (2.6%)	9 (2.1%)	24 (2.4%)	
Medium	160 (27.3%)	104 (24.1%)	264 (26%)	
Higher	406 (69.3%)	316 (73.3%)	722 (71%)	
Sex of the child				
Boy	291 (49.7%)	242 (56.1%)	533 (52.4%)	*p* = 0.0702 * Phi = 0.057
Girl	295 (50.3%)	189 (43.9%)	484 (47.6%)	
Total	586 (57.6%)	431 (42.4%)	1017 (100%)	
Age of children surveyed in months	Q1 = 11.0	Q1 = 9.0	Q1 = 10.0	*p* < 0.0001 ** r = −0.133
	Me = 16.0	Me = 13.0	Me = 12.0	
	Q3 = 18.0	Q3 = 17.0	Q3 = 18.0	
Body weight of the examined children [kg]	Q1 = 9.5	Q1 = 9.0	Q1 = 9.0	*p* = 0.0022 * r = −0.098 *
	Me = 10.5	Me = 10.0	Me = 10.0	
	Q3 = 12.0	Me = 11.5	Q3 = 12.0	
Height/body length of children tested [kg]	Q1 = 78.0	Q1 = 75.0	Q1 = 76.0	*p* < 0.0001 ** r = −0.129
	Me = 82.0	Me = 80.0	Me = 82.0	
	Q3 = 87.0	Q3 = 86.0	Q3 = 86.0	

Q1—25th percentile, Q3—75th percentile, Me—median. * Chi-squared test. ** Mann–Whitney U test. Cramér’s V = Cr.V.

**Table 2:** Characteristics of feeding and introduction of complementary foods in children with BLW and NoBLW N = 1017 (100%).

		BLW	NoBLW	Total	*p*-Value Chi^2^ Test
		N = 586	N = 431	N = 1017	
Feeding method in the first months of life	Exclusive breastfeeding	404	253	657	*p* = 0.0007 Cr.V = 0.105
		68.90%	58.70%	64.60%	
	Feeding with milk formula	350	309	659	*p* < 0.0001 Cr.V = 0.124
		59.70%	71.70%	64.80%	
Length of breastfeeding	Was not fed breast milk at all	29	33	62	*p* = 0.000 Cr.V = 0.059
		4.90%	7.70%	6.10%	
	Child was not fed with breast milk; only at the hospital was the baby given colostrum	27	47	74	
		4.60%	10.90%	7.30%	
	Up to 1 month of age	56	40	96	
		9.60%	9.30%	9.40%	
	1–2 months	34	47	81	
		5.80%	10.90%	8.00%	
	3–4 months	58	38	96	
		9.90%	8.80%	9.40%	
	5–6 months	35	35	70	
		6.00%	8.10%	6.90%	
	6–12 months	70	40	110	
		11.90%	9.30%	10.80%	
	13–24 months	65	21	86	
		11.10%	4.90%	8.50%	
	I continue to feed	209	129	338	
		35.70%	29.90%	33.20%	
	I don’t remember	3	1	4	
		0.50%	0.20%	0.40%	
Time of introduction of milk formula (if the baby was fed milk formula)	On day 1 of life	106	104	210	*p* = 0.07
		18.10%	24.10%	20.60%	
	Up to 4 months of age	105	86	191	
		17.90%	20%	18.80%	
	Up to 6 months of age	23	20	43	
		3.90%	4.60%	4.20%	
	Between 6 and 12 months of age	48	36	84	
		8.20%	8.30%	8.30%	
	After 1 year of age	17	12	29	
		2.90%	2.80%	2.90%	
	In hospital, after the birth	87	64	151	
		14.80%	14.80%	14.90%	
	Was not fed milk formula	200	109	309	
		34.10%	25.30%	30.40%	
Initiation of complementary feeding	Before 4 months of age	0	17	17	*p* = 0.006 Cr.V = 0.058
		0	3.94%	1.67%	
	Between 4 and 5 months of age	112	93	205	
		19.11%	21.58%	20.16%	
	Between 6 and 7 months of age	468	321	789	
		79.86%	74.48%	77.58%	
	Between 8 and 12 months of age	6	0	6	
		1.02%	0.00%	0.59%	
	After 12 months of age	0	0	0	
		0	0	0	

**Table 3:** Comparison of first food introduction and complementary feeding methods in children with BLW and NoBLW method, N = 1017 (100%).

	BLW N = 586	NoBLW N = 431	Total N = 1017	*p*-Value Chi^2^ Test
The first product to be introduced during complementary feeding				
Green vegetables	130	65	195	*p* = 1.00
	22.2%	15.1%	19.2%	
Pumpkin	109	95	204	
	18.6%	22%	20.1%	
Carrot	242	194	436	
	41.3%	45%	42.9%	
Potato	49	30	79	
	8.4%	7%	7.8%	
Porridge	7	16	23	
	1.2%	3.7%	2.3%	
Apple	18	12	30	
	3.1%	2.8%	2.9%	
Pear	1	1	2	
	0.2%	0.2%	0.2%	
Meat	0	0	0	
	0.0%	0.0%	0.0%	
Sugar	0	0	0	
	0.0%	0.0%	0.0%	
Use of pudding, pudding with lumps, and solids during complementary feeding				
Pudding *	511	421	932	*p* =0.000 Cr.V = 0.211
	87.2%	97.7%	91.64%	
Pudding with lumps **	526	411	937	*p* =0.001 Cr.V = 0.100
	89.8%	95.34%	92.13%	
Products that the child can take into his or her own hands	579	363	942	*p* =0.000 Cr.V = 0.222
	98.8%	84.2%	92.63%	

* pudding—e.g., fruit mousse, yoghurt; ** pudding with lumps—e.g., fruit mousse with small pieces of fruit, yoghurt with oatmeal.

**Table 4:** Periods of introduction of individual foods during complementary feeding using BLW (BLW—N = 586; 100%) and NoBLW (NoBLW—N = 431; 100%).

	BLW/ NoBLW	Before 4 Months of Age	Between 4 and 6 Months of Age	Between 6 and 12 Months of Age	After 12 Months of Age	I Have Not Introduced	I Don’t Remember	*p*-Value
Vegetables	BLW	10	189	368	0	0	1	*p* = 0.006
	N = 586	(1.7%)	(32.3%)	(65.9%)	(0.0%)	(0.0%)	(0.2%)	
	No BLW	7	185	238	0	1	0	
	N = 431	(1.6%)	(42.9%)	(55.2%)	(0.0%)	(0.2%)	(0.0%)	
Fruit	BLW	8	169	405	2	1	1	*p* = 0.01
	N = 586	(1.4%)	(28.8%)	(69.1%)	(0.3%)	(0.2%)	(0.2%)	
	No BLW	6	167	252	1	4	1	
	N = 431	(1.4%)	(38.7%)	(58.5%)	(0.2%)	(0.9%)	(0.2%)	
Hen’s eggs	BLW	3	61	471	26	21	4	*p* = 0.003
	N = 586	(0.5%)	(10.4%)	(80.4%)	(4.4%)	(3.6%)	(0.7%)	
	No BLW	0	47	326	15	41	2	
	N = 431	(0.0%)	(10.9%)	(75.6%)	(3.5%)	(9.5%)	(0.5%)	
Gluten	BLW	2	81	468	15	16	4	*p* = 0.0005
	N = 586	(0.3%)	(13.8%)	(79.9%)	(2.6%)	(2.7%)	(0.7%)	
	No BLW	0	66	311	10	36	8	
	N = 431	(0.0%)	(15.3%)	(72.2%)	(2.3%)	(8.4%)	(1.9%)	
Peanuts *	BLW	0	13	259	81	227	6	*p* = 0.0004
	N = 586	(0.0%)	(2.2%)	(44.2%)	(13.8%)	(28.7%)	(1%)	
	No BLW	0	7	141	51	228	4	
	N = 431	(0.0%)	(1.6%)	(32.7%)	(11.8%)	(52.9%)	(0.9%)	
Fat	BLW	2	97	441	29	14	3	*p* = 0.0008
	N = 586	(0.3%)	(16.6%)	(75.3%)	(4.9%)	(2.4%)	(0.5%)	
	No BLW	0	72	297%	22	34	6	
	N = 431	(0.0%)	(16.1%)	(68.9%)	(5.1%)	(7.9%)	(1.4%)	
Meat	BLW	0	104	462	8	10	2	*p* = 0.73
	N = 586	(0.0%)	(17.7%)	(78.8%)	(1.4%)	(1.7%)	(0.3%)	
	No BLW	0	86	326	9	9	1	
	N = 431	(0.0%)	(20.0%)	(75.6%)	(2.1%)	(2.1%)	(0.2%)	
Meat preparations	BLW	1	41	228	129	175	12	*p* = 0.078
	N = 586	(0.2%)	(7.0%)	(38.9%)	(22.0%)	(29.9%)	(2.0%)	
	No BLW	0	25	164	73	163	6	
	N = 431	(0.0%)	(5.8%)	(38.1%)	(16.9%)	(37.8%)	(1.4%)	
Fish	BLW	0	75	454	28	25	4	*p* = 0.001
	N = 586	(0.0%)	(12.8%)	(77.5%)	(4.8%)	(4.3%)	(0.7%)	
	No BLW	0	62	302	20	46	1	
	N = 431	(0.0%)	(14.4%)	(70.1%)	(4.6%)	(10.7%)	(0.2%)	
Cereal products	BLW	2	115 (19.6%)	455	11	2	1	*p* = 0.00003
	N = 586	(0.3%)		(77.6%)	(1.9%)	(0.3%)	(0.2%)	
	No BLW	3	97	295	15	20	1	
	N = 431	(0.7%)	(22.5%)	(68.4%)	(3.5%)	(4.6%)	(0.2%)	
Salt	BLW	0	11	116	242 (41.3%)	206	11	*p* = 0.17
	N = 586	(0.0%)	(1.9%)	(19.8%)		(35.2%)	(1.9%)	
	No BLW	2	13	88	151	168	9	
	N = 431	(0.5%)	(3.0%)	(20.4%)	(35.0%)	(39.0%)	(2.1%)	
Sweetened beverages	BLW	2	6	54	93	410	21	*p* = 0.48
	N = 586	(0.3%)	(1.0%)	(9.2%)	(15.9%)	(70.0%)	(3.6%)	
	No BLW	0	3	53	70	288	17	
	N = 431	(0.0%)	(0.7%)	(12.3%)	(16.2%)	(66.8%)	(3.9%)	
Honey	BLW	1	5	53	197	311	19	*p* = 0.045
	N = 586	(0.2%)	(0.9%)	(9.0%)	(33.6%)	(53.1%)	(3.2%)	
	No BLW	1	4	44	103	263	16	
	N = 431	(0.2%)	(0.9%)	(10.2%)	(23.9%)	(61.0%)	(3.7%)	
Water	BLW	59 (10.1%)	159 (27.1%)	351	13	4	0	*p* = 0.006
	N = 586			(59.9%)	(2.2%)	(0.7%)	(0.0%)	
	No BLW	64 (14.8%)	144 (33.4%)	211	6	5	1	
	N = 431			(49.0%)	(1.4%)	(1.2%)	(0.2%)	
Cow’s milk	BLW	1	33	291	117	128	16	*p* < 0.0001
	N = 586	(0.2%)	(5.6%)	(49.7%)	(20.0%)	(21.8%)	(2.7%)	
	No BLW	0	22	160	81	154	14	
	N = 431	(0.0%)	(5.1%)	(37.1%)	(18.8%)	(35.7%)	(3.2%)	
Sheep’s milk	BLW	2	6	60	25	442	51	*p* = 0.376
	N = 586	(0.3%)	(1.0%)	(10.2%)	(4.3%)	(75.4%)	(8.7%)	
	No BLW	0	5	39	24	337	26	
	N = 431	(0.0%)	(1.2%)	(9.0%)	(5.6%)	(78.2%)	(6.0%)	
Goat’s milk	BLW	2	8	69	29	429	49	*p* = 0.69
	N = 586	(0.3%)	(1.4%)	(11.8%)	(4.9%)	(73.2%)	(8.4%)	
	No BLW	2	5	44	22	332	26	
	N = 431	(0.5%)	(1.2%)	(10.2%)	(5.1%)	(77.0%)	(6.0%)	
Fruit juices	BLW	0	17	65	133	335	36	*p* = 0.116
	N = 586	(0.0%)	(2.9%)	(11.1%)	(22.7%)	(57.2%)	(6.1%)	
	No BLW	2	22	56	84	247	20	
	N = 431	(0.5%)	(5.1%)	(13.0%)	(19.5%)	(57.3%)	(4.6%)	
Plant-based drinks	BLW	1	20	199	70	261	35	*p* = 0.01
	N = 586	(0.2%)	(3.4%)	(34.0%)	(11.9%)	(44.5%)	(6.0%)	
	No BLW	1	17	110	38	240	25	
	N = 431	(0.2%)	(3.9%)	(25.5%)	(8.8%)	(55.7%)	(5.8%)	

* in the form of peanut butter/peanut flour.

**Table 5:** Rating of occurrence of vomiting reflex, spitting out food, gagging, choking, and choking with medical intervention by respondents, N = 586 (100%).

		Answer	Number of Respondents N = 586	
			N	%
Problems experienced during BLW	Vomiting reflex	yes	277	47.3%
	Spitting out food	yes	452	77.1%
	Gagging	yes	380	64.8%
	Choking	yes	72	12.3%
	Choking with medical intervention	yes	1	0.2%
Period of introduction of the BLW method by mothers	Between 4 and 5 months	yes	16	2.7%
	Between 6 and 7 months	yes	355	60.6%
	Between 8 and 12 months	yes	205	35.0%
	After 12 months	yes	10	1.7%
Form of food used in the BLW method	Soups	yes	359	61.3%
	Pulps, purees	yes	368	62.8%
	Tubes	yes	478	81.6%
	Strips	yes	495	84.5%
	Cubes	yes	359	61.3%
	Posts	yes	557	95.1%
Was your child spoon-fed before the introduction of BLW?		yes	429	73.2%
When using the BLW method, could the child decide what to eat?		yes	543	92.7%
When using the BLW method, could the child decide how much to eat?		yes	585	99.8%
Has the child been fed with a spoon?		yes	478	81.6%
Does the child eat on their own with a spoon?		yes	414	70.7%
Does the child use their hands to eat by themselves?		yes	579	98.1%

**Table 6:** Results of linear and logistic regression models analysing the association between BLW, the child’s age, and their weight status.

Model	Variable	Beta Coefficient	*p*-Value	95% CI (Lower)	95% CI (Upper)
Linear regression	BLW/NoBLW	−0.02634	0.75802	−0.19408	0.14139
	Child’s Age	0.22698	0.0	0.21279	0.24116
Logistic regression	BLW/NoBLW	0.07084	0.59546	−0.19066	0.33233
	Child’s Age	0.01904	0.09701	−0.00345	0.04152

## Data Availability

The data presented in this study are available on request from the corresponding author. The data are not publicly available due to restrictions that apply to the availability of these data.
